# A systematic approach to parameter selection for CAD-virtual reality data translation using response surface methodology and MOGA-II

**DOI:** 10.1371/journal.pone.0197673

**Published:** 2018-05-23

**Authors:** Mustufa Haider Abidi, Abdulrahman Al-Ahmari, Ali Ahmad

**Affiliations:** 1 Princess Fatima Alnijiris’s Research Chair for Advanced Manufacturing Technology (FARCAMT Chair), Advanced Manufacturing Institute, King Saud University, Riyadh, Saudi Arabia; 2 Industrial Engineering Department, College of Engineering, King Saud University, Riyadh, Saudi Arabia; 3 Louisiana Community and Technical College System-Manufacturing Extension Partnership, Baton Rouge, LA, United States of America; King Abdulaziz University, SAUDI ARABIA

## Abstract

Advanced graphics capabilities have enabled the use of virtual reality as an efficient design technique. The integration of virtual reality in the design phase still faces impediment because of issues linked to the integration of CAD and virtual reality software. A set of empirical tests using the selected conversion parameters was found to yield properly represented virtual reality models. The reduced model yields an R-sq (pred) value of 72.71% and an R-sq (adjusted) value of 86.64%, indicating that 86.64% of the response variability can be explained by the model. The R-sq (pred) is 67.45%, which is not very high, indicating that the model should be further reduced by eliminating insignificant terms. The reduced model yields an R-sq (pred) value of 73.32% and an R-sq (adjusted) value of 79.49%, indicating that 79.49% of the response variability can be explained by the model. Using the optimization software MODE Frontier (Optimization, MOGA-II, 2014), four types of response surfaces for the three considered response variables were tested for the data of DOE. The parameter values obtained using the proposed experimental design methodology result in better graphics quality, and other necessary design attributes.

## Introduction

Virtual reality (VR) integrates powerful digital computers, with special software and hardware to establish a digital environment. The aim of VR is to create reality that is persuasive as real and interactive to the user [1]. VR technologies have been implemented in the product design process in various industrial sectors, including automotive, aerospace, defense, and bio-engineering [2, 3]. For industries involving intricate products, it is critical not only to design the geometrical shapes of the product parts using computer-aided design (CAD) packages, but also to assess the interior and exterior characteristics prior to building the first prototype. With the aid of advanced visualization systems, VR offers a platform for the design review process. Immersive visualization using tools such as; a large display and stereoscopic vision, or a head-mounted display (HMD) can enhance the users’ understanding of innovative products.

In the field of automotive, for example, VR has been used to visualize digital mock-ups of new cars at a real-life scale [4]. Compared with CAD software, current VR systems offer remarkable capabilities that can augment the visualization of 3D design models. Graphical tools are an integral part of the design process, and 92% of communications are graphically based on VR systems [5]. Hence, design and the decision making process can be enhanced by using the effective graphics conveyance tools such as, VR [6, 7].

By virtue of novel navigation techniques, the design engineer is able to design and interact with a virtual model in a more instinctive and competent way. VR-based systems have enabled significant achievements in the manufacturing field. They have been implemented in many manufacturing domains, particularly in the areas of product design, assembly, and maintenance [8].

One of the obstacles to use VR in the design process is the problem of integration between CAD tool kits, and VR-based systems. In contrast to CAD software, which is desktop-based, VR systems use HMDs and high-resolution 3D displays, which yield a far superior spatial understanding of the models. The primary cause of data translation problems is, the different internal mathematical schemes used to represent objects that arise from the accuracy norms used to estimate curvatures, trajectories, and facets. To run practical VR simulations (such as those for visualization and collision detection), rational CAD models must be transformed into tessellated ones [9]. A major challenge faced by VR practitioners is the integration of the various hardware and software components, associated with VR systems.

The CAD to VR data translation process involves the conversion of Non-uniform Rational B-Spline (NURBS) surfaces from the CAD model to the model of polygonal for use in VR. Hence, a tessellation procedure is needed to transform the NURBS surfaces into triangular ones. The parameters implicated in the triangulation process affect the polygonal model results. Such a triangulation process can be realized, using various techniques based on different approaches and algorithms. A CAD model can be transformed into VR either directly or through a third-party rendering package. In general, there are three distinct classes of methods for CAD-VR data conversion: library-based methods, database methods, and straightforward translation methods [9].

This mitigates the time spent on repetitive tasks, but requires an immense initial effort to create the library. In a database approach, a central database is used by both the CAD software and the virtual environment to retrieve a part of information. Finally, straight-forward conversion processes are popular methods for transforming CAD data into VR. In such a process, an entire CAD model is transformed into VR either directly or through some intermediary tool, which necessitates a certain exchange interface.

This can be accomplished by exporting CAD data in neutral formats such as the Initial Graphics Exchange Specification (IGES), the Standard for the Exchange of Product Model Data (STEP), or extensible Mark-up Language (XML), or by converting the data into a VR-compatible format using intermediate converters or adapters. To convert complex CAD data, standard graphical data exchange formats are used, such as STEP, IGES, and XML [9].

The lack of interoperability between Virtual reality (VR) and CAD systems is particularly due to the problem of efficiently importing the data of CAD into VR simulation. It helps in minimizing the loss of information. The different internal mathematics representation schemes are the major problem of translating the data from CAD system to VR reality. The problem of accuracy criteria arises due to differences in performing calculation, curves, and surfaces. In order to perform real-time VR simulation, the CAD models must be converted into tessellated ones. Moreover, once the conversion of files is completed, all the information and results will be lost that are specifically related to the reciprocal conversion, and physical characteristics of the model [9].

In the data translation process from CAD model to VR, several problems have been detected regarding the models visualization. Geometry and precision are the common losses in the process of data translation. Moreover, the model of CAD includes, some project information that is linked to each object. During the translation of CAD to VR, the system lost this information that is linked with the object [9].

The representation of shape and interoperability of product models have been presented by Lee [10]. This model focused on the distribution of virtual prototyping, and used to transfer and share product models [10]. Other formats have also been used to convert information from CAD models into VR systems, such as OpenFlight, the Drawing Exchange Format (DXF), and 3D Studio (3DS) format. Weyrich and Drew described a method called a “virtual workbench” for virtual assembly. The system used the Multigen Creator commercial tool of modelling [11].

Bourdot et al. [12] introduced an approach for combining VR with CAD, in which they instituted a VR-CAD framework with multimodal immersive interaction. Tang and Gu developed a novel approach for transforming CAD data into a native VR format, using a reduction algorithm [13]. Yap et al. presented a generic approach of converting CAD data for virtual manufacturing using Open GL and the lexical analyzer generator Lex. Their system can successfully convert data between four file formats: stereo lithography (STL), VRML, XML, and object [14]. Lorenz et al. [15], presented a CAD and VR system self-determining workflow for an automated CAD model complexity reduction, and furthermore the developed methodology preservs the animation and kinematic mechanism.

The Design of Experiment (DOE) is as statistical technique that is used for the optimization of systems’ performance with known input variables. DOE is a systematic method that is used for the identification of a relationship between the factors that affect the outcomes of that process. In the context of product design, DOE is used for determining the optimal parameters which are helpful in the translation of data from CAD to VR model. The two major parameters that are used for data translation are Tolerance and chord length; however, DOE is a major tool used for the selection of optimized parameter during the translation process [14].

Tolerance indicates the maximum time, by which tessellation is permitted to lose from the systematic surface (deviation or error). The maximum distance between the tessellated surface and original design is known as Chord length or chordal tolerance [14]. The majority of research on CAD-to-VR data conversion has been conducted, using the data translation interface technique. Translation interfaces are provided by several commercial software suites (such as Deep Exploration and Product View) and CAD systems (CATIA, Pro/E, etc.).

Previous literature indicates that there is a lack of interoperability between CAD and VR, because of the inefficiency of importing CAD data into VR simulations with minimal information loss. Many studies as mentioned above have addressed the data translation issue of CAD and VR systems, but most have focused on the loss of attribute information. Less attention has been paid to the loss of geometric information, which also plays an imperative role in the development of a realistic virtual environment. The objective of the study is based on the appropriate selection of parameters for the translation of data from CAD to VR model. Previous research papers [3, 9, 12] have focused on the problems of integration between CAD-VR data translation, but this study is merely focused on the development of a systematic experimental design methodology to select the parameters to be used in the CAD-VR data translation.

## Materials and methods

**Fig 1:**
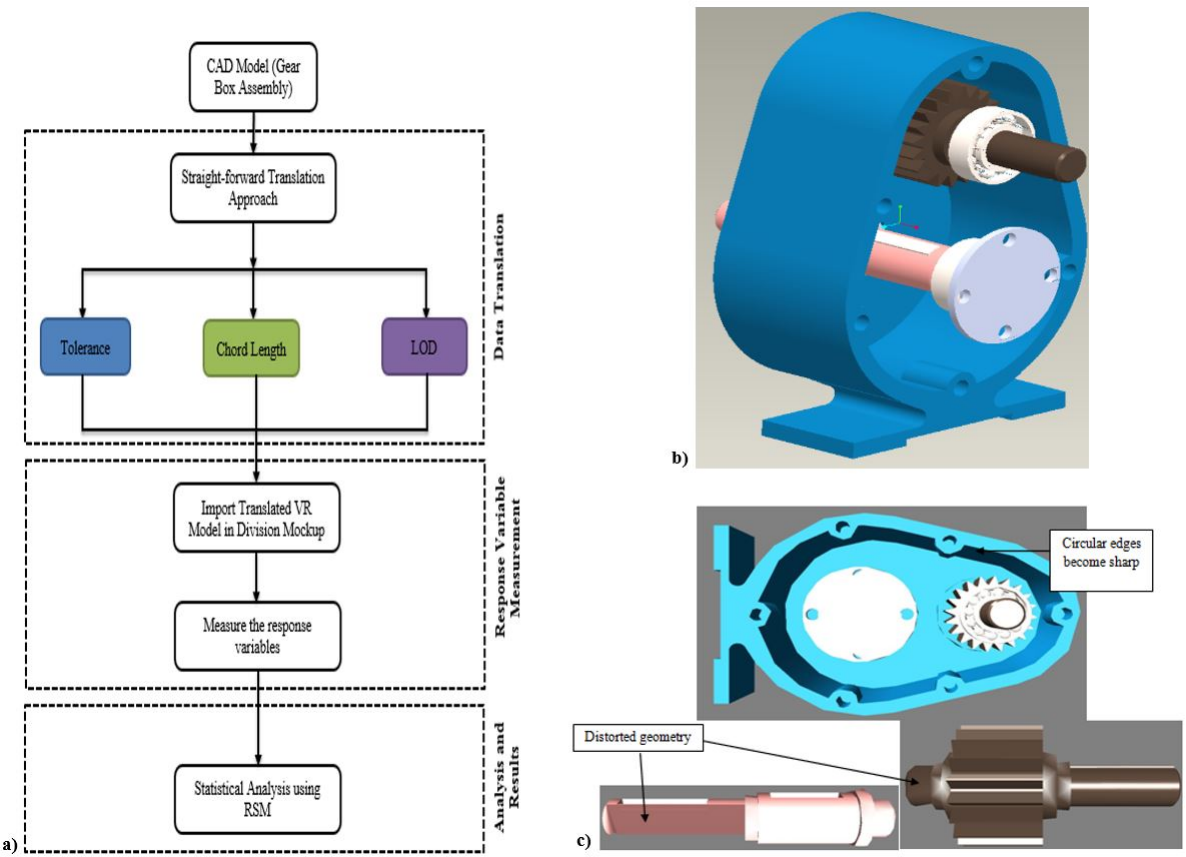
a) Methodology for the selection of translation parameters b) Gearbox assembly model from CAD c) Converted VR model with translation problems.

The Design of Experiments (DOE) technique was applied in this study, to determine the appropriate parameters to be used in translation procedure. Fig 1 shows a block diagram of the steps of the implemented procedure. It represents the methodology for the selection of parameters for the translation process. This flow chart is comprised on five steps. In the first step, it is needed to select an appropriate gearbox assembly for the experiment. After selecting the gearbox, the straight-forward translation approach is used to import the data in to translate VR Model, in the division Mock-up. The translation parameters defined in Fig 1 are Tolerance, Chord Length, and LOD detail. The imported data is then transferred for measuring the responses of variables. A complete statistical analysis has been undertaken by using the response surface strategy (RSM) [16].

The selection of parameter is significantly affecting the output responses; therefore, the best selected technique for optimizing the parameter is, DOE technique. There are seven basic steps involved in implying the DOE technique to any experiment or process. The steps are as follows: problem recognition, selection of factors, setting ranges and levels, selection of response variables, selection of experimental design, assessing the experiment performance, analysis of data, and conclusions and recommendations [16].

The steps of DOE are highly interdependent on each other. If wrong problem is recognized in the first step, then the whole process of DOE fails to develop the desired results. Therefore, it is necessary to clearly undertake each step to select appropriate parameters for the conversion process. In step two, the selection of factors and their levels are important for identifying the characteristics of performance of any process or product. The third step is interrelated with the second step because it is based on the selection of responses of the desired variables. It is also helpful in assessing the process performance. In fourth step, full analysis and factorial design has been selected. The measurement of the selected design and statistical analysis of the result has been identified in the fifth and sixth step. The last step is based on the conclusion and recommendation of the whole process. These steps were useful for the CAD-VR data translation procedure [16].

### Problem recognition

A gearbox assembly model for CAD was chosen as the subject of the experiments. This assembly is appropriate for this purpose because the components of a gearbox include various geometric characteristics, such as; rectangular, circular, and freeform shapes. Fig 1 also shows the gearbox assembly model for CAD.

Straightforward translation approach was used to transfer the CAD data into the VR format. Product View adapters were selected as the middle interface for the conversion of the CAD model from the PTC Pro-e format to the PTC Division Mockup 2000i^2^ format. Many parameters are associated with Product View adapters, and these parameters must be properly selected to ensure high-quality results. An inappropriate selection of conversion parameters can lead to problems in the transformed VR model; for example, the model circular edges may not appear smooth, or the overall geometry may appear imprecise. Fig 1 further illustrates some of the problems that can arise in an improperly converted VR model [17].

### Selections of levels, factors, and ranges

The performance characteristics of any product or process can be evaluated by selecting the appropriate parameters, and their levels and ranges. Therefore, it is essential to properly select the experiments’ factors and levels. This requires a careful study of the system. In order to identify the important factors, different preliminary experiments were performed. Based on the results of these experiments, three parameters were identified, as summarized in Table 1.

**Table 1:** Values and levels of the most important parameters.

Factors	Number of Levels	Values
Tolerance (millimeters)	2	0.01 and 0.03
Chord Length (millimeters)	2	0.1 and 0.5
Number of Levels of Detail (LODs)	2	3 and 7

#### Tolerance

Tolerance indicates the maximum time by which tessellation is permit to lose from the systematic surface (deviation or error).

#### Chord length

The maximum distance between the tessellated surface and original design is known as, Chord length or chordal tolerance.

#### Number of Levels of Detail (LODs)

The LOD concept invokes the idea that when the spectator is distant from the object, not all details in the file must be shown or drawn.

#### Selection of response variables

The correct response variable(s) selection for testing is crucial because, the performance of the process is assessed through these variables. The selected response variables for the CAD-VR translation process are specified in Table 2.

**Table 2:** Selection of response variables for CAD-VR translation.

Response	Unit
Graphics Quality	Very Good (3), Good (2) and Poor (1)
File Size	Kilobytes (KB)
Rendering Time	Seconds
Number of Triangles	Number

#### Graphics quality

The graphics quality represents the quality of the output model in the environment of VR, i.e., how good it looks, and is considered to be the most imperative response. All the graphics quality are rated on the basis of how clear and sharp image it present in the environment of VR. The quality of very good, shows that there is a very sharp image and graphics present in the output model of VR; whereas, the poor quality represent the poor image effects of the output of VR model. Graphics quality is also rated on the basis of final representation of the VR models. Textures, illumination and reflection models have also been considered before ranking the quality of graphics. This is a subjective measure, and it is determined by averaging the responses of ten users based on their experience and then rounding off the resulting value.

#### File size

The file size is the space in the memory of computer, in units of KB, required by the converted model. A relatively moderate model was selected for this case study, a file size in KB is adequate. The model size MB can also be used in other different experiments; such as, a complete car, units of megabytes (MB) would be appropriate.

#### Rendering time

The time required by the computer to render the complete model in the VR environment. The whole experiment has been conducted by using the same computer and with the same load condition.

#### Number of triangles

The total number of triangles contained in the transformed model of polygonal.

### Choice of experimental design and analysis

Response surface methodology (RSM) was selected as the design of experiment technique to be used in this study. The optimal values of the translation parameters were numerically determined using the RSM quadratic model. To save time and effort, it is desirable to reduce the number of periods without affecting the desired objectives. The analysis of model and problem can be helpful through RSM, which is a statistical technique used to influence the certain variables and objectives in order to optimize the responses [16]. To achieve this purpose, strategies such as central composite design (CCD) in RSM are frequently used [18]. The total number of runs for this study was calculated as follows: 8 cube points, 6 center points in each cube, and 6 axial points. The face-cantered design was selected; therefore, α = 1.

### Apparatus

The gearbox assembly model of CAD was used to perform the experiment. The following are the specifications that computer had to perform: an Intel Core 2 Quad Q9400 @ 2.66 GHz processor with 4 GB of RAM and an NVIDIA GeForce 9800GT graphics card with 1 GB of memory.

### Procedure

The Division Mockup 2000i^2^ software tool was used to measure the variable responses. Table 3 summarizes the RSM CCD for the selected factors [19].

**Table 3:** RSM CCD table.

Factors				Responses			
Run Order	Tolerance	Chord Length	Number of LODs	No. of Triangles	File Size (KB)	Rendering Time (sec)	Graphics Quality
1	0.01	0.1	3	96158	1540	0.4	3
2	0.03	0.1	3	83804	1340	0.36	2
3	0.01	0.5	3	74708	887	0.3	1
4	0.03	0.5	3	68646	801	0.22	1
5	0.01	0.1	7	95142	1527	0.37	3
6	0.03	0.1	7	82876	1323	0.33	2
7	0.01	0.5	7	74235	872	0.25	1
8	0.03	0.5	7	67983	793	0.21	1
9	0.01	0.3	5	92567	1453	0.35	2
10	0.03	0.3	5	72345	835	0.28	1
11	0.02	0.1	5	87658	1400	0.37	3
12	0.02	0.5	5	69752	829	0.26	1
13	0.02	0.3	3	72638	866	0.28	2
14	0.02	0.3	7	71865	847	0.24	1
15	0.02	0.3	5	72273	852	0.26	1
16	0.02	0.3	5	72273	852	0.26	1
17	0.02	0.3	5	72273	852	0.26	1
18	0.02	0.3	5	72273	852	0.26	1
19	0.02	0.3	5	72273	852	0.26	1
20	0.02	0.3	5	72273	852	0.26	1

## Results and discussion

### Response surface methodology

To evaluate the influence of the preferred factors on the responses, RSM analysis was performed using the Minitab 17 software package. Based on RSM, the central composite rotatable second-order design was used to conduct the experiment. The mathematical relationship between the response (Z_j_) and the various translation parameters were established through the response surface modelling, with the aim of identifying the optimal settings for data translation [20]. The influence of parameter on various response criteria was analyzed by using general second-order polynomial response surface mathematical model, the various response criteria are as follows [16]:

$$Z_{j}=\beta_{0}+\sum_{i=1}^{k}\beta_{i}x_{ij}+\sum_{i=1}^{k}\beta_{ii}x_{ij}^{2}+\sum_{i<l}\beta_{il}x_{ij}x_{lj}+e_{j}$$ (1)

Here, Z_j_ is the response, x_ij_ is the coded value of the ith translation parameter of the j^th^ experiment, k is the number of translation parameters, β_i_, β_ii_, and β_il_ are the second-order regression coefficients, and the residual e_j_ is a measure of the experimental error on the j^th^ observation. RSM was used to investigate which factor had the strongest effect on which response. In addition, the effects on the responses of interactions between the factors were also examined.

#### Number of triangles

An ANOVA table was constructed to summarize the test for significance of the regression model, the tests for significance of the individual model coefficients and the lack-of-fit test (Table 4).

**Table 4:** ANOVA table for number of triangles.

Source	DF	Adj. SS	Adj. MS	F-Value	P-Value
Model	9	1470481560	163386840	20.61	0.000
Linear	3	1143827254	381275751	48.09	0.000
Tolerance	1	326680834	326680834	41.21	0.000
Chord Length	1	815661860	815661860	102.89	0.000
LOD	1	1484561	1484561	0.19	0.674
Square	3	307641693	102547231	12.94	0.001
Tolerance*Tolerance	1	139420280	139420280	17.59	0.002
Chord Length*Chord Length	1	31217996	31217996	3.94	0.075
LOD*LOD	1	26159259	26159259	3.3	0.099
2-Way Interaction	3	19012613	6337538	0.8	0.522
Tolerance*Chord Length	1	18929704	18929704	2.39	0.153
Tolerance*LOD	1	1301	1301	0	0.990
Chord Length*LOD	1	81608	81608	0.01	0.921
Error	10	79278868	7927887		
Lack-of-Fit	5	79278868	15855774	-	-
Pure Error	5	0	0		
Total	19	1549760428			
Model Summary S R-sq R-sq(adj) R-sq(pred) 2815.65 94.88% 90.28% 67.45%					

In ANOVA table, the variation in different components of the model have been measured by the values of adjusted sum square. The adjusted mean square indicates that how much variation a term or model explain, despite the order they were entered in the table. The test output reveals that the response of quadratic model is significant. The study used 95% of the significance level.

The goodness of fit was evaluated using R^2^ (coefficient of correlation) values to check the consistency and accuracy of the model. The ANOVA results indicate that Tolerance and Chord Length have a significant effect (p-value < 0.05) on the number of triangles. The result from table 4 indicates that, tolerance and chord length are the two major parameters that are used for the creation of new triangles. The quadratic term Tolerance^2^ also has a significant effect. The quadratic term Chord Length^2^ may have a slight effect on the response, as its p-value (0.075) is close to 0.05. The R-sq (pred) is 67.45%, which is not very high, indicating that the model should be further reduced by eliminating insignificant terms. After the removal of the insignificant terms, the reduced model for Number of Triangles is as follows:

NumberofTriangles=127699−3488080Tolerance−45157ChordLength+72913000Tolerance*Tolerance (2)

This reduced model yields an R-sq (pred) value of 85.05% and an R-sq (adjusted) value of 89.15%, indicating that 89.15% of the response variability can be explained by the obtained model. The R-sq (pred) value is used to determine how well the model can predict new observations.

**Fig 2:**
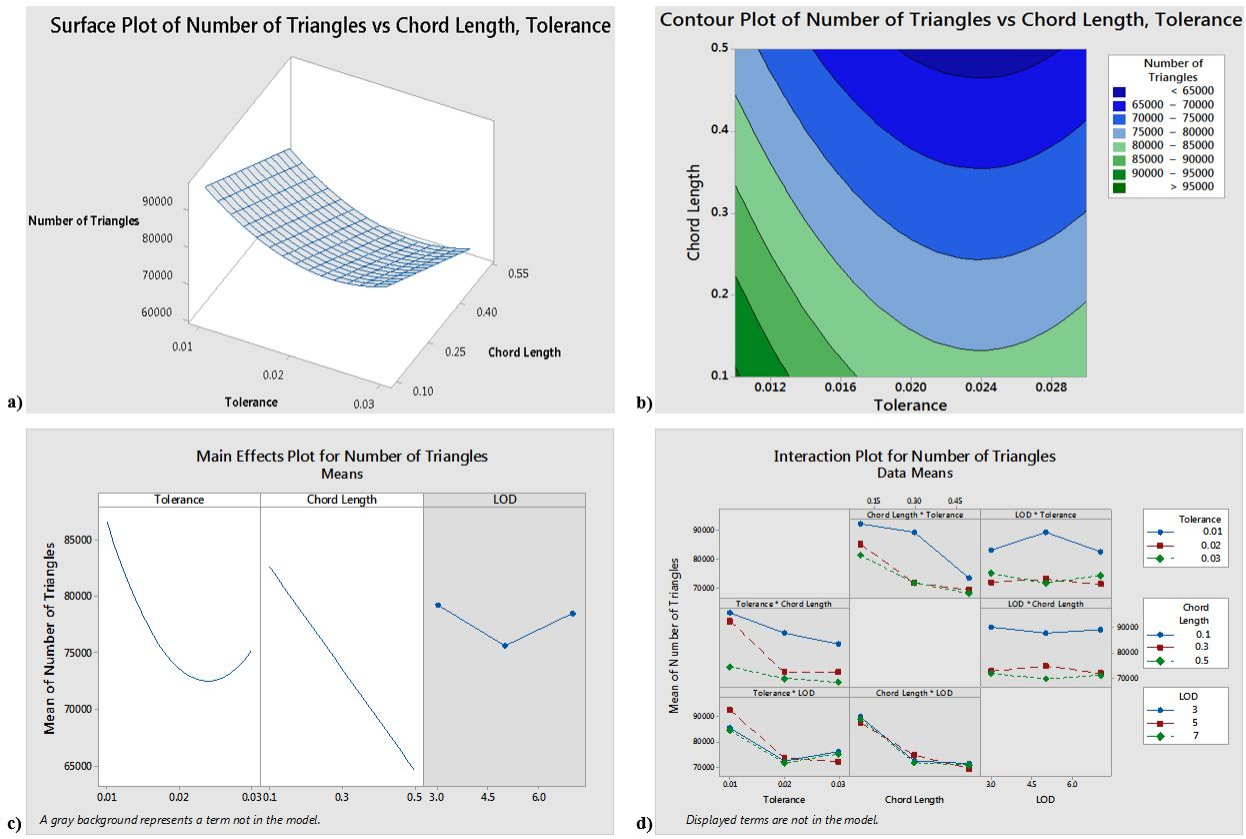
a) Responses surface for Number of Triangles b) Contour plot for Number of Triangles c) Main effects plot for Number of Triangles d) Interaction plot for Number of Triangles.

Fig 2 shows that to obtain the minimum number of triangles, the tolerance should be approximately 0.024 and the chord length should be approximately 0.5. It further shows that tolerance and Chord Length are the significant factors, and that a tolerance of 0.024 and a chord length of 0.5 will result in the smallest number of triangles. The interaction plot suggests that some of the interactions may be significant; however, the ANOVA results reject the significance of the interaction terms.

#### File size

The ANOVA results for File Size are given in Table 5.

**Table 5:** ANOVA table for file size.

Source	DF	Adj. SS	Adj. MS	F-Value	P-Value
Model	9	1362364	151374	12.21	0.000
Linear	3	1010486	336829	27.18	0.000
Tolerance	1	140897	140897	11.37	0.007
Chord Length	1	869070	869070	70.13	0.000
LOD	1	518	518	0.04	0.842
Square	3	344731	114910	9.27	0.003
Tolerance*Tolerance	1	101136	101136	8.16	0.017
Chord Length*Chord Length	1	72414	72414	5.84	0.036
LOD*LOD	1	25200	25200	2.03	0.184
2-Way Interaction	3	7147	2382	0.19	0.899
Tolerance*Chord Length	1	7140	7140	0.58	0.465
Tolerance*LOD	1	1	1	0	0.993
Chord Length*LOD	1	6	6	0	0.983
Error	10	123930	12393		
Lack-of-Fit	5	123930	24786	-	-
Pure Error	5	0	0		
Total	19	1486294			
Model Summary S R-sq R-sq(adj) R-sq(pred) 111.324 91.66% 84.16% 43.26%					

The test output reveals that the response of quadratic model is significant. The study used 95% of the significance level. The ANOVA results indicate that Tolerance and Chord Length have a significant effect (p-value < 0.05) on the file size. The quadratic terms Tolerance^2^ and Chord Length^2^ also have a significant effect. The R-sq (pred) value is 43.26%, which is low, indicating that the model should be further reduced by eliminating insignificant terms. After the removal of the insignificant terms, the reduced model for File Size is as follows:

FileSize=2468−74220Tolerance−3370ChordLength+1558750Tolerance*Tolerance+3159ChordLength*ChordLength (3)

**Fig 3:**
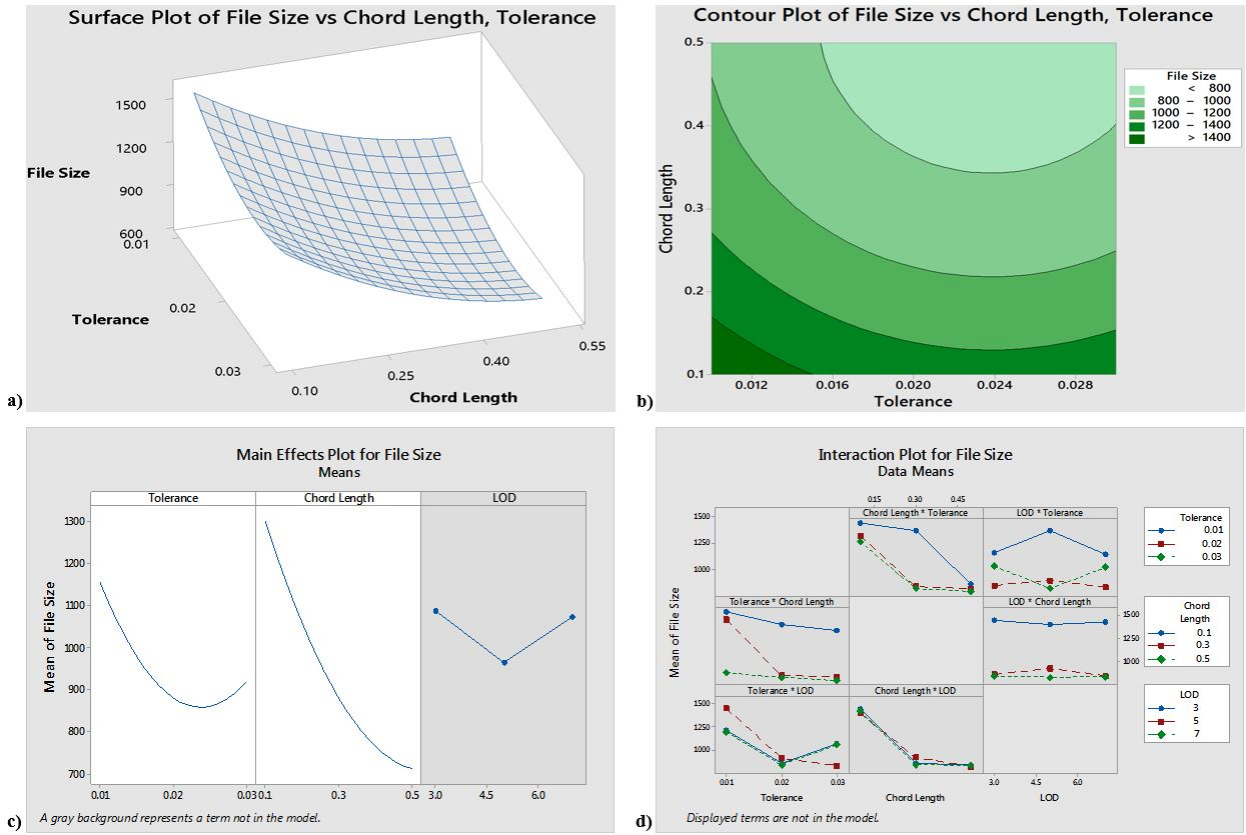
a) Responses surface for File Size b) Computer plot for File Size c) Main effects for File Size d) Interaction for File Size.

The reduced model yields an R-sq (pred) value of 72.71% and an R-sq (adjusted) value of 86.64%, indicating that 86.64% of the response variability can be explained by the model. The units in the axes of the plots are chord length (millimeters), tolerance (millimeters) and file size (KB). Fig 3 shows that, to obtain the minimum file size, the tolerance should be approximately 0.024 and the chord length should be approximately 0.4. Fig 3 has further shown that the tolerance of 0.024 and a chord length of 0.4 which results in the smallest file size. The interaction plot suggests that some of the interactions may be significant; again, however, the ANOVA results reject the significance of the interaction terms.

#### Rendering time

The ANOVA results for Rendering Time are given in Table 6.

**Table 6:** ANOVA table for rendering time.

Source	DF	Adj. SS	Adj. MS	F-Value	P-Value
Model	9	0.054696	0.006077	26.61	0.000
Linear	3	0.04466	0.014887	65.19	0.000
Tolerance	1	0.00729	0.00729	31.92	0.000
Chord Length	1	0.03481	0.03481	152.43	0.000
LOD	1	0.00256	0.00256	11.21	0.007
Square	3	0.009636	0.003212	14.07	0.001
Tolerance*Tolerance	1	0.002705	0.002705	11.85	0.006
Chord Length*Chord Length	1	0.002705	0.002705	11.85	0.006
LOD*LOD	1	0.001536	0.001536	6.73	0.027
2-Way Interaction	3	0.0004	0.000133	0.58	0.639
Tolerance*Chord Length	1	0.0002	0.0002	0.88	0.371
Tolerance*LOD	1	0.0002	0.0002	0.88	0.371
Chord Length*LOD	1	0	0	0	1.000
Error	10	0.002284	0.000228		
Lack-of-Fit	5	0.002284	0.000457	-	-
Pure Error	5	0	0		
Total	19	0.05698			
Model Summary S R-sq R-sq(adj) R-sq(pred) 0.0151117 95.99% 92.39% 75.02%					

The test output reveals that the response of quadratic model is significant. The study used 95% of the significance level. The ANOVA results indicate that Tolerance, Chord Length, and LOD all have a significant effect (p-value < 0.05) on the rendering time. The quadratic terms Tolerance^2^, Chord Length^2^, and LOD^2^ also has a significant effect. The R-sq (pred) value is 75.02%, which is high; however, it can be further improved by eliminating insignificant terms. After the removal of the insignificant terms, the reduced model for Rendering Time is as follows:

RenderingTime=0.5003−15.25Tolerance−0.765ChordLength+0.0511LOD+313.6Tolerance*Tolerance+0.784ChordLength*ChordLength−0.00591LOD*LOD (4)

**Fig 4:**
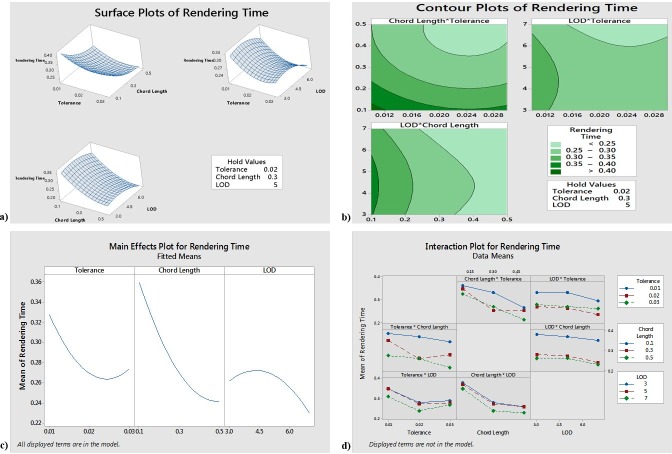
a) Response surfaces for Rendering Time b) Contour plots for Rendering Time c) Main effects for Rendering Time d) Interaction plot for Rendering Time.

The reduced model yields an R-sq (pred) value of 85.44% and an R-sq (adjusted) value of 93.12%, indicating that 93.12% of the response variability can be explained by the model. Fig 4 indicated that, to obtain the minimum rendering time, the tolerance should be approximately 0.024, the chord length should be approximately 0.4, and the LOD should be 6. Fig 4 further showed that Tolerance, Chord Length, and LOD are the significant factors for Rendering Time and that a tolerance value of 0.024, a chord length of 0.4, and LOD value of 6 will result in the shortest rendering time. The interaction plots suggest that some of the interactions may be significant; however, the ANOVA results reject the significance of the interaction terms.

#### Graphics quality

The ANOVA results for Graphics Quality are summarized in Table 7.

**Table 7:** ANOVA table for graphics quality.

Source	DF	Adj. SS	Adj. MS	F-Value	P-Value
Model	9	9.7182	1.0798	8.42	0.001
Linear	3	7.4	2.46667	19.24	0.000
Tolerance	1	0.9	0.9	7.02	0.024
Chord Length	1	6.4	6.4	49.93	0.000
LOD	1	0.1	0.1	0.78	0.398
Square	3	1.8182	0.60606	4.73	0.026
Tolerance*Tolerance	1	0.0057	0.00568	0.04	0.837
Chord Length*Chord Length	1	0.8182	0.81818	6.38	0.030
LOD*LOD	1	0.0057	0.00568	0.04	0.837
2-Way Interaction	3	0.5	0.16667	1.3	0.328
Tolerance*Chord Length	1	0.5	0.5	3.9	0.077
Tolerance*LOD	1	0	0	0	1.000
Chord Length*LOD	1	0	0	0	1.000
Error	10	1.2818	0.12818		
Lack-of-Fit	5	1.2818	0.25636	-	-
Pure Error	5	0	0		
Total	19	11			
Model Summary S R-sq R-sq(adj) R-sq(pred) 0.358025 88.35% 77.86% 31.78%					

The test output reveals that the response of quadratic model is significant. The study used 95% of the significance level. The ANOVA results indicate that Tolerance and Chord Length have a significant effect (p-value < 0.05) on the graphics quality. The quadratic term Chord Length^2^ also has a significant effect. The R-sq (pred) value is 31.78%, which is very low, indicating that the model should be further reduced by eliminating insignificant terms. After the removal of the insignificant terms, the reduced model for Graphics Quality is as follows:

GraphicsQuality=4.350−30.0Tolerance−13.00ChordLength+15.00ChordLength*ChordLength (5)

**Fig 5:**
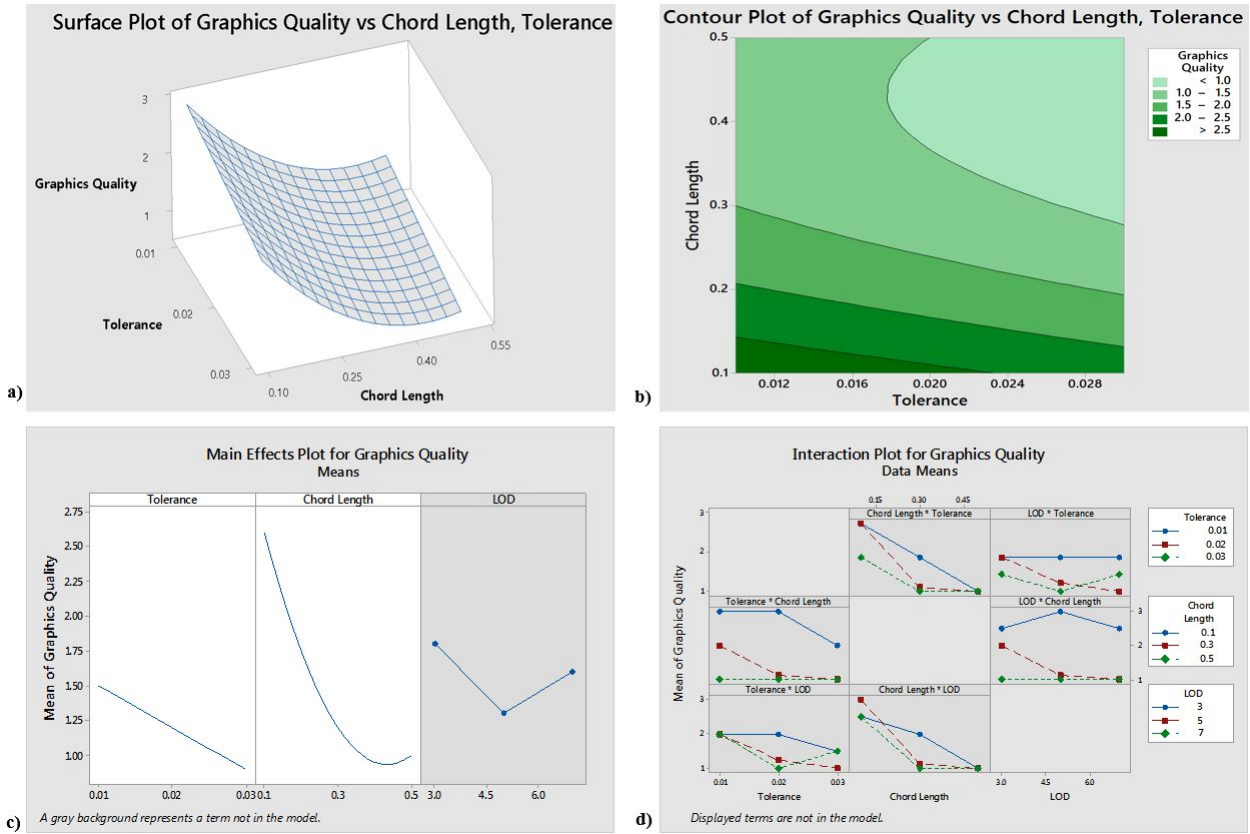
a) Response surface for Graphics Quality b) Contour plot for Graphics Quality c) Main effects plot for Graphics Quality d) Interaction plot for Graphics Quality.

The reduced model yields an R-sq (pred) value of 73.32% and an R-sq (adjusted) value of 79.49%, indicating that 79.49% of the response variability can be explained by the model. Fig 5 indicated that to obtain the maximum graphics quality, the tolerance should be approximately 0.012 and the chord length should be approximately 0.1. Fig 5 further indicated that Tolerance and Chord Length are significant factors, and that a tolerance of 0.01 and a chord length of 0.1 will result in the best graphics quality. The interaction plot suggests that some of the interactions may be significant; however, the ANOVA results reject the significance of the interaction terms.

### Model validation

**Fig 6:**
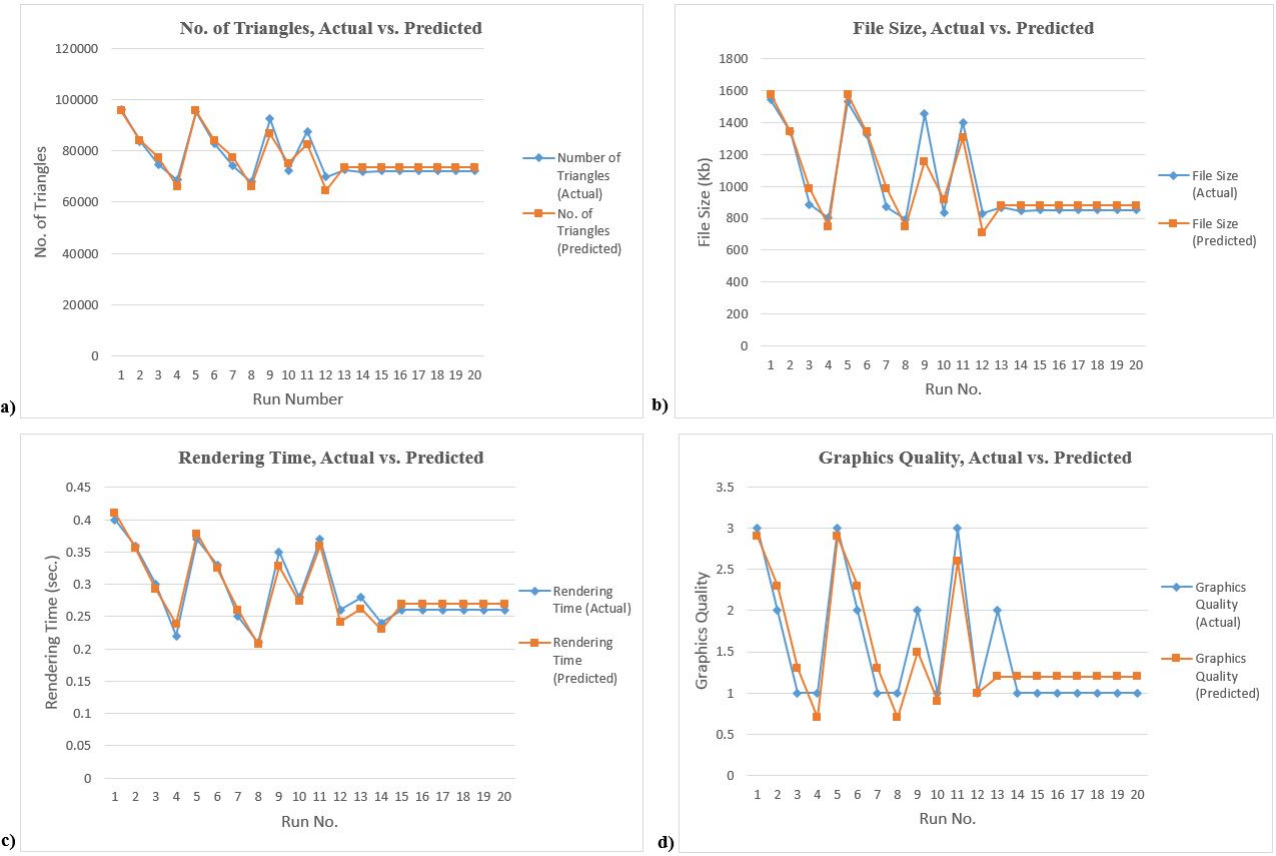
a) Actual vs. predicted values for Number of Triangles b) Actual vs. predicted values for File Size c) Actual vs. predicted values for Rendering Time d) Actual vs. predicted values for Graphics Quality.

To evaluate the obtained model, a graphical comparison was generated for each of the four responses. Fig 6 has shown the graphical comparisons of the actual versus predicted values for all four responses. Fig 6 further depicted that the predicted values of the obtained models are very close to the actual response values.

### Multi-objective optimization

The results for the four responses; Number of Triangles, File Size, Rendering Time, and Graphics Quality are shown in Table 3. In the multi-objective optimization, the number of triangles was not considered because it is directly correlated with the file size.

**Fig 7:**
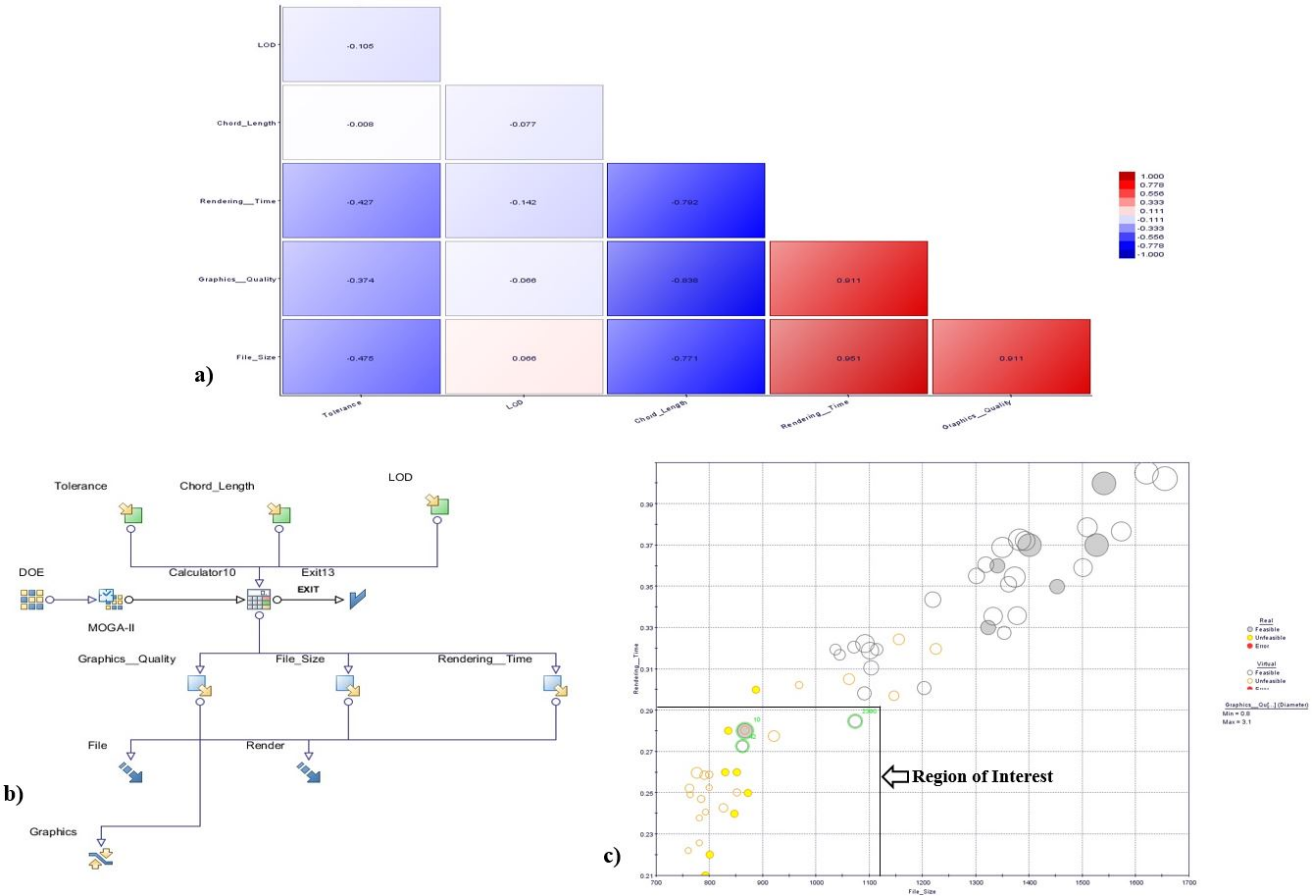
a) Matrix of correlations between inputs and responses b) Optimization workflow using MOGA-II and RSM c) 3D bubble chart obtained in the optimization search.

Fig 7 shows the matrix of the correlations between the various factors and responses involved in the translation process. Fig 7 also shows that there are strong relationships between File Size and Rendering Time, between Graphics Quality and File Size, and between Graphics Quality and Rendering Time. Certain pairs of parameters have inverse relationships; such as, that between File Size and Tolerance, and that between Graphics Quality and Tolerance.

Using the optimization software MODE Frontier, four types of response surfaces for the three considered response variables were tested for the data of DOE. The four types of surfaces were as follows: two polynomial models, one of degree 1 and one of degree 2; a model of neutral network, and a radial basis function (RBF) model. The comparison of response surface model was illustrated in Table 8.

**Table 8:** Comparison of response surface models for file size.

RSM parameter	Polynomial model, degree 1	Polynomial model, degree 2	Neural network model	Radial basis function model
Mean absolute error	1.11E02	6.38E01	8.31E0	3.33E-13
Mean relative error	1.16E-1	6.19E-2	8.01E-3	3.84E-16
Mean normalized error	1.49E-1	8.54E-2	1.11E-2	4.46E-16
R-squared	7.80E-1	9.19E-1	9.98E-1	1.00E0

The error of the RBF model is negligible compared with that of the other models because the RBF yields inter-polant surfaces that exceed precisely through the points of training. Therefore, the response surface based on the RBF model was chosen for the optimization tool.

The aim of the study was to determine the best likely input parameters for attaining good quality of graphics while minimizing the size of file and rendering time. The problem of multi-objective optimization was invented to minimize the file size and rendering time while setting a lower limit on the graphics quality. Table 9 summarizes the objective function and the applied constraint. Fig 7 also depicts the optimization workflow that was developed using MODE Frontier.

**Table 9:** Objective function and constraints for the optimization study.

Objective Function	1. Minimize File Size
	2. Minimize Rendering Time
Constraint	Graphics Quality ≥ 1.5

MOGA-II is an enhanced form of the Multi-Objective Genetic Algorithm (MOGA) [21]. Moreover, MOGA-II is highly user-friendly because it requires the user to specify only a few parameters; all other parameters are established internally. The initial population was created using the DOE table. The total number of evaluations attempted by the algorithm is equal to the product of the initial population and the number of generations. From the beginning of the design matrix, a total of 100 generation was run.

Fig 7 shows a bubble chart of the results obtained in the optimization search using MOGA-II. The chart was generated by plotting the file size and rendering time on the X and Y axes, respectively. The graphics quality, i.e., the constraint parameter, is represented by the size of the bubble. Points that exist in the DOE design matrix are regarded as real points, whereas the virtual points are those predicted via RSM. Because the objective is to minimize both file size and rendering time, the lower left quadrant is the region of interest; i.e., the optimal design points lie in this region.

Real points are represented by filled bubbles, and virtual points, by hollow bubbles. Yellow bubbles represent unfeasible design points and red bubbles represent error points. Three points (labelled 1, 2, and 3) in the lower left quadrant were identified as optimal design points. Of these, design point 2 is based on reality, corresponding to design 10 in the original matrix of DOE.

**Fig 8:**
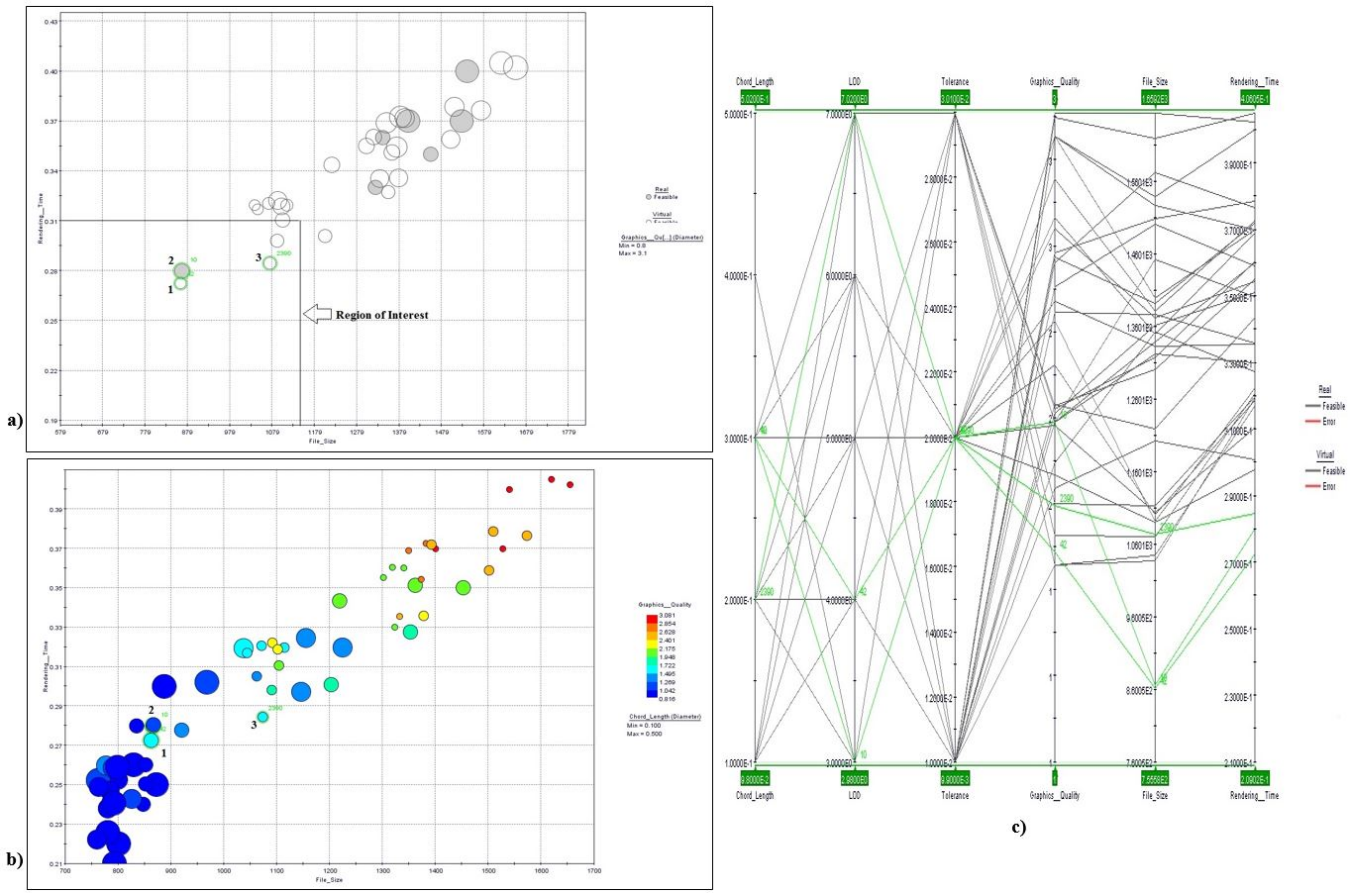
a) Bubble chart of feasible points b) 4D bubble chart obtained in the optimization search c) Parallel coordinate chart including only feasible points.

**Fig 9:**
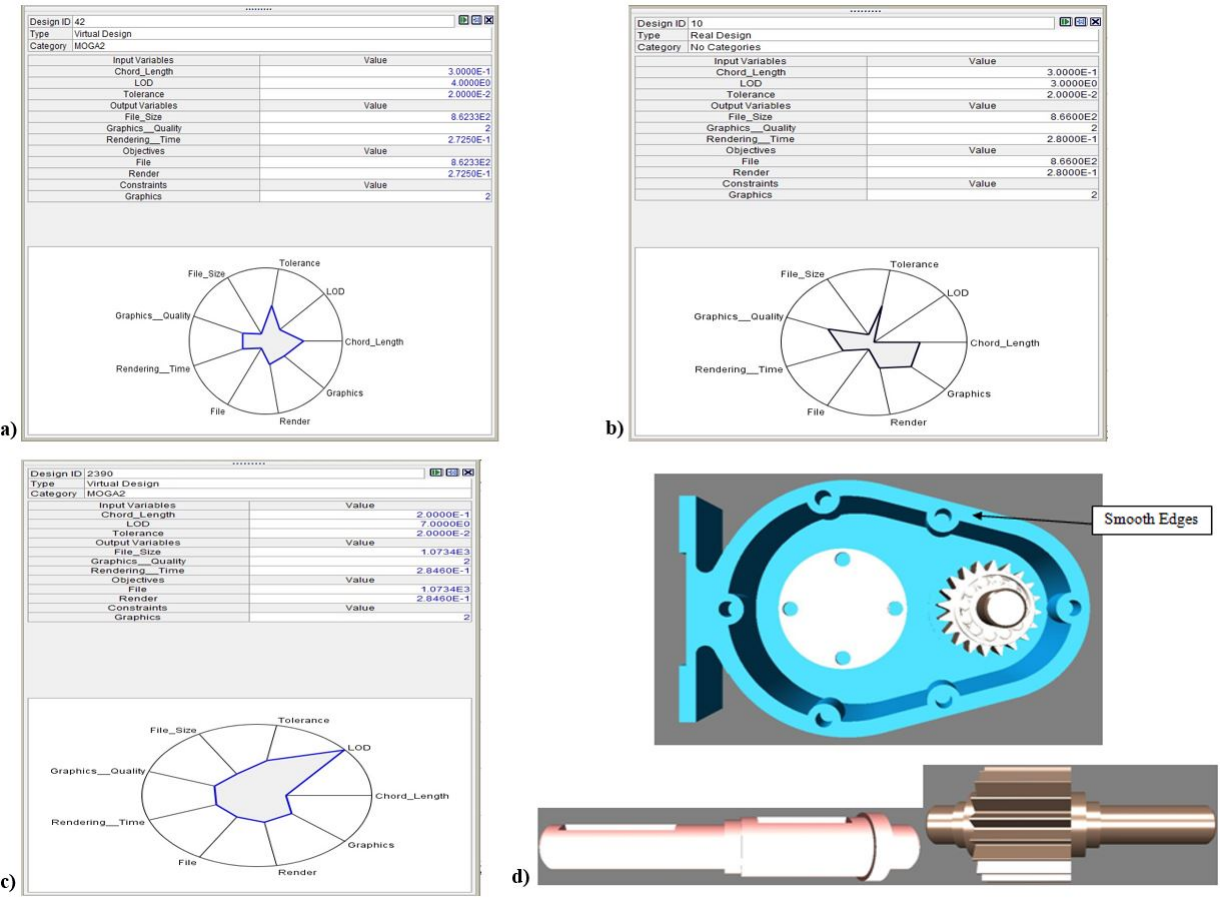
a) Optimal solution at Point 1 (design point 42) b) Optimal solution at point 2 (design point 10) c) Optimal solution at point 3 (design point 2390) d) VR model of the gearbox assembly obtained using the recommended parameters.

Fig 8 shows a version of the bubble chart that contains only the feasible points. Points 1, 2, and 3 are considered to be the optimal design points. Of these, point 2 is real and also has the largest diameter, indicating that it corresponds to the best graphics quality. A clearer view is provided in Fig 8, which adds chord length information to yield a 4D bubble chart. Fig 8 indicates that point 1 will result in better graphics quality with a smaller file size and a shorter rendering time. Fig 8 also shows a parallel coordinate chart of the input parameters and responses, including only the feasible points. Fig 9 shows the best solutions obtained at Point 1, Point 2, and Point 3, respectively. Table 10 summarizes the details of the optimal solutions from Fig 9.

**Table 10:** Details of the optimal solutions.

ID	Tolerance	Chord Length	LOD	File Size (KB)	Rendering Time (sec)	Graphics Quality
Point1	0.02	0.3	4	862.33	0.2725	2
Point2	0.02	0.3	3	866	0.280	2
Point3	0.02	0.2	7	1073.4	0.2850	2

Table 10 shows that all optimal solutions correspond to a moderate tolerance and chord length. Point 2 (design point 10) is a real point; hence, it can be selected as an optimal solution.

The lack of incorporation between the VR and CAD software engaged in the design process means that significant effort is required to prepare a realistic virtual environment [22]. Although, the inter-operability between VR systems and CAD software has been improved, and considerable attention has been focused on this area, there is still no standard procedure or format available for CAD-to-VR data conversion. In a previous study by one of the authors [23], the conversion problem was statistically addressed using full factorial methods, but the CCD RSM and multi-objective optimization approaches were not applied. Therefore, the models developed in that study did not reflect the quadratic nature of the responses.

Based on this study, the following conclusions are presented:

- A statistical approach in selecting the appropriate conversion parameters was proven to be effective for the development of realistic, high-quality virtual environments.
- The impacts of CAD-VR translation parameters were evaluated to identify the parameters’ optimal value; thus, to attain the best quality of graphics with the minimization of file size, number of triangles and rendering time. The translation parameters that most influence the quality of graphics were found to be the tolerance and chord length. Except the rendering time, the number of LODs was found to have least influence on all responses.
- The study findings suggest the following values are recommended for diverse CAD-VR translation parameters to achieve better quality of graphics:
  Tolerance=0.01,ChordLength=0.1,andNumberofLODs=3. (6)
- The parameters recommended above result in good quality of graphics but also increase the file size, number of triangles, and rendering time. Therefore, multi-objective optimization techniques will be applied in future work.
- The model of CAD contain elements of lesser geometrical complication, that do not necessitate a large number of triangles in their tessellated models but still yield a high quality of graphics in their VR models. Therefore, with simple features of CAD models, the recommended set of parameters are as follows:
  Tolerance=0.03,ChordLength=0.1,andNumberofLODs=7. (7)
- The performance of multi-objective optimization was determined with the optimal values of the translation parameters that would yield the best result in combination with the applied constraint. The results obtained in this study are as follows:
  TheoptimaldesignpointisPoint2,withTolerance=0.02,ChordLength=0.3,andNumberofLODs=3. (8)

Therefore, multi-objective optimization provides accurate, high-quality results. This procedure enables the selection of the design point that will result in the best response for a chosen parameter, while maintaining the other responses at an acceptable level. The VR model that is obtained by relevant suggested parameters for the translation of the selected CAD model (i.e., the gearbox assembly) are shown in Fig 9.

**Fig 10:**
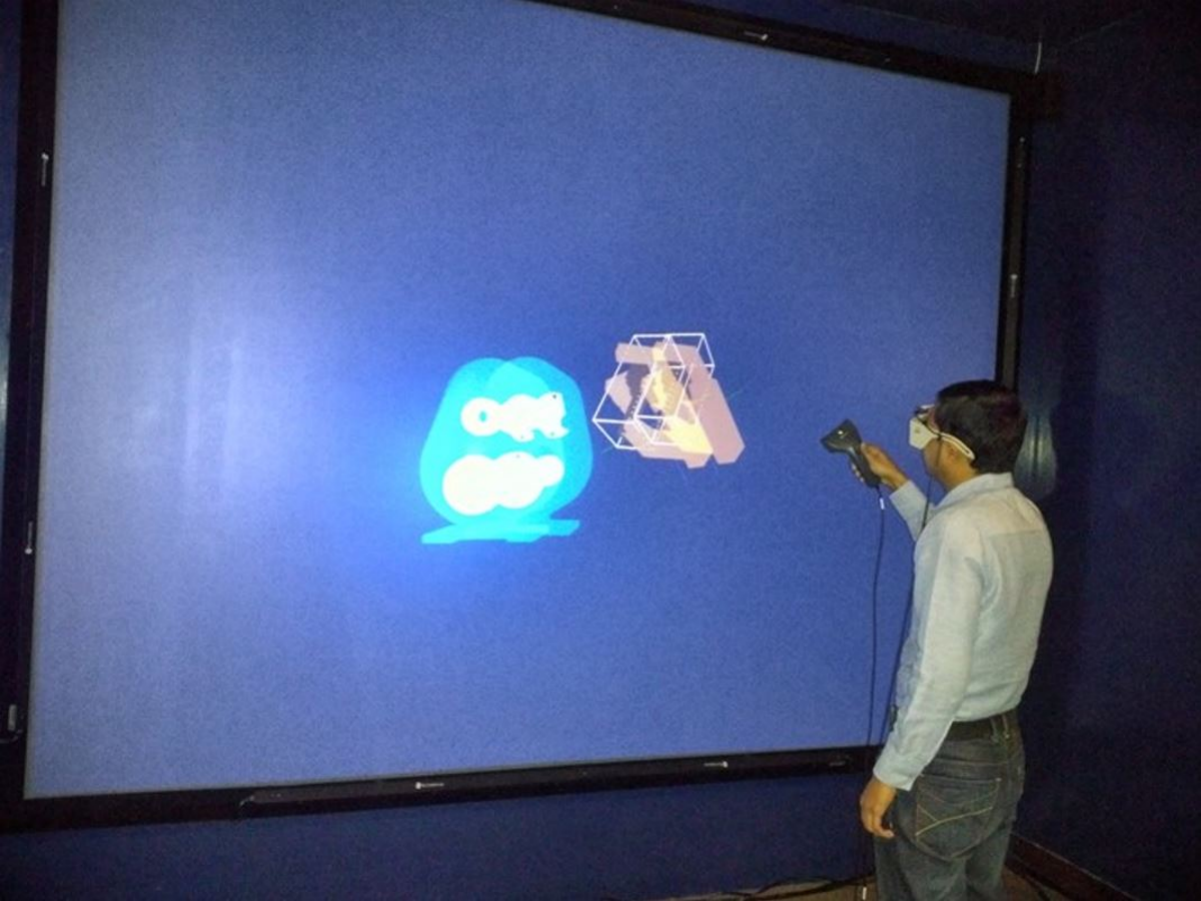
A user analyzing the gearbox model in a semi-immersive virtual environment.

The results obtained using the proposed methodology, can be applied for the development of auto-stereoscopic systems. The statistical approach presented in this study extends the technique of straight forward CAD-VR data translation [24], and provides a new perspective on the optimization of the conversion process. Fig 10 shows a user, analyzing the final gearbox assembly in a semi-immersive virtual environment by using ahead and hand tracker. All previous studies have been conducted on identifying the problems related to the integration of CAD-VR data translation. This study provides a new insight by providing new methods of optimization of parameters, which are helpful in the data translation from CAD to VR model. Moreover, the problem of information loss in the data translation can also be reduced by using the optimize conversion process of data translation.

## Conclusions

This study concludes that, while triangulation of a surface, the chord length and tolerance are precisely the two parameters that are used for the creation of a new triangle; therefore, the resulting triangulation is directly related to these two parameters. Moreover, both tolerance and chord length are the dependent variables that are used for the creation of a number of triangles. This study also concludes that new triangles are directly correlated with the size of the file.

In future, a more comprehensive study must be conducted, based on the optimization and generation of a predictive mathematical model using RSM. Additional case studies at different levels of complexity will be considered to examine, whether the results are model-specific or dependent on particular model characteristics, such as size and complexity. The author had limited expertise with the utilization of commercial products; therefore, further research can be conducted by utilizing more products of PTC.
